# Exploring the role of natural bioactive molecules in genitourinary cancers: how far has research progressed?

**DOI:** 10.1007/s13659-023-00400-4

**Published:** 2023-10-16

**Authors:** Fahadul Islam, Nikhil Nath, Mehrukh Zehravi, Jishan Khan, Sumiya Ben-Ta Jashim, Manoj Shrawan Charde, Rita Dadarao Chakole, K. Praveen Kumar, A. Kishore Babu, Firzan Nainu, Sharuk L. Khan, Safia Obaidur Rab, Talha Bin Emran, Polrat Wilairatana

**Affiliations:** 1 Department of Pharmacy, Faculty of Allied Health Sciences, Daffodil International University, Dhaka, 1207 Bangladesh; 2 Department of Pharmacy, International Islamic University Chittagong, Kumira, Chittagong, 4318 Bangladesh; 3 Department of Clinical Pharmacy, College of Dentistry & Pharmacy, Buraydah Private Colleges, Buraydah, 51418 Kingdom of Saudi Arabia; 4 Government College of Pharmacy, Vidyanagar, Karad, Satara, 415124 Maharashtra India; 5 Department of Pharmaceutical Chemistry, School of Pharmaceutical Sciences, Govt. of NCT of Delhi, Delhi Pharmaceutical Sciences and Research University (DPSRU), Mehrauli-Badarpur Road, PushpVihar, Sector 3, New Delhi, 110017 India; 6 Ratnadeep College of Pharmacy, Ratnapur, Jamkhed, Ahmednagar, 413206 Maharashtra India; 7 Department of Pharmacy, Faculty of Pharmacy, Hasanuddin University, Makassar, 90245 Indonesia; 8 Department of Pharmaceutical Chemistry, N.B.S. Institute of Pharmacy, Ausa, 413520 Maharashtra India; 9 Department of Clinical Laboratory Sciences, College of Applied Medical Sciences, King Khalid University, Abha, Saudi Arabia; 10 Department of Pathology and Laboratory Medicine, Warren Alpert Medical School & Legorreta Cancer Center, Brown University, Providence, RI 02912 USA; 11 Department of Clinical Tropical Medicine, Faculty of Tropical Medicine, Mahidol University, Bangkok, 10400 Thailand

**Keywords:** Genitourinary cancers, Apoptosis, Urinary tract, Chemotherapy, Polyphenols

## Abstract

The primary approaches to treat cancerous diseases include drug treatment, surgical procedures, biotherapy, and radiation therapy. Chemotherapy has been the primary treatment for cancer for a long time, but its main drawback is that it kills cancerous cells along with healthy ones, leading to deadly adverse health effects. However, genitourinary cancer has become a concern in recent years as it is more common in middle-aged people. So, researchers are trying to find possible therapeutic options from natural small molecules due to the many drawbacks associated with chemotherapy and other radiation-based therapies. Plenty of research was conducted regarding genitourinary cancer to determine the promising role of natural small molecules. So, this review focused on natural small molecules along with their potential therapeutic targets in the case of genitourinary cancers such as prostate cancer, renal cancer, bladder cancer, testicular cancer, and so on. Also, this review states some ongoing or completed clinical evidence in this regard.

## Introduction

Cancers of the urinary tract (kidney, bladder, ureter, and urethra) and male reproductive organs (testes, testicular tissue, prostate, and penis) are called genitourinary cancers. Gynecologic malignancies, often known as cancers of the female reproductive system, damage a woman's genitalia and ovaries. Genitourinary cancers are lethal, making them the most deadly [1]. Numerous therapeutic classes are being developed to treat genitourinary cancers, including mTOR inhibitors of tyrosine kinases with small molecules and unnatural fusion proteins. In the case of metastatic bladder cancer (MBC), Erdafitinib was the first Food and Drug Administration (FDA)-approved targeted therapy. In addition, the FDA recently authorized immune checkpoint inhibitors in combination with targeted treatments [2]. Bladder cancer (BC) is a prevalent malignancy that affects individuals of both genders, ranking as the fourth most frequently occurring form of cancer. Both sexes are equally at risk for developing the fourth most frequent form of bladder cancer. There is a broad spectrum of severity in BC, from lazy, slowly progressing tumors to aggressive, rapidly progressing tumors with high mortality rates and a need for intrusive monitoring over a long period [3]. Small cell carcinoma (less than 0.5%), adenocarcinoma (0.5–2%), squamous cell carcinoma (3–5%), carcinosarcoma/sarcomatoid tumors (sarcoma, sarcoma, melanoma, paraganglioma, and lymphoma) are all rare forms of cancer (less than 0.1%) [4]. Two types of BC: papillary tumors that invade the muscles. And those that do not and are non-papillary (solid) muscle-invasive tumors. The clinical and molecular features of the two subtypes are unique [5]. In 2018, there were a total of 1,276,106 new cases of prostate cancer diagnosed, which resulted in 358,989 fatalities. Adenocarcinomas encompass the majority of prostate tumors and share many features with other common epithelial malignancies, such as colon and breast cancer [6, 7]. Among males, renal cell carcinomas (RCCs) rank ninth most often diagnosed malignancy, while among women, it rank fourteenth [8]. Von Hipple-Lindau (VHL) disease, a dominantly inherited cancer syndrome, is the most frequent cause of hereditary RCC. 70% of patients with VHL develop renal cell carcinoma by age 60 [9]. The eight primary tumors and cell types (entities) with distinct morphologic features used for the current classification of renal cell adenomas (RCAs) and carcinomas are as follows: For example, there are eight different types of RCCs: (1) clear-cell RCCs, (2) chromophobe-cell RCAs/RCCs, (3) chromophobic RCAs/RCCs, (4) duct Bellini RCCs, (5) transitional-cell RCCs, (6) neuroendocrine RCCs, (7) oncocytic RCAs, and (8) metal RCCs [10]. An orphan illness, penile squamous cell carcinoma (PSCC), affects just 0.1–1 in every 100,000 males in developed countries. However, in certain parts of Asia, Africa, and South America, it still causes up to 10% of male malignancies [11] because early diagnosis and treatment of penile cancer require a high level of suspicion for the disease and a minimum threshold for biopsy of penile lesions that demonstrate short-term resistance to conservative therapy [12]. The most commonly observed etiology of malignant urethral obstruction is plasmacytoid urethral carcinoma (PUC) [13]. Although uncommon, urethral carcinoma is the only genitourinary cancer affecting females disproportionately. In males, the urethra is much longer and more intricate than the urethra in females [14]. Among the genitourinary cancers (bladder, prostate, testicular, and renal cell), the average risk decreases when comparing high vs. low levels of physical activity is less than 10%, according to a review of epidemiologic studies of physical activity and genitourinary malignancies [15]. BC is one of several illnesses for which smoking is known to increase the likelihood of development. Arsenic exposure from drinking water at quantities more than 300 g/l is another risk factor for developing BC. Occupational exposure to 4,4′-methylenebis(2-chloroaniline) and aromatic amines, found in the chemical, rubber industries, and dye, as well as fungicides, cigarette smoke, hair dyes, plastics, paints, motor vehicle exhaust, and metals, are the risk factors for developing BC [4]. The human papillomavirus (HPV) and phimosis are major contributors to penile cancer [16]. In addition to these, other risk factors include sexually transmitted diseases, smoking, lack of personal cleanliness, and poverty. There's a lot of evidence indicating smoking increases the chances of getting testicular cancer [17].

Most drugs, including aspirin, insulin, and antihistamines, are small molecules. Along with main metabolites like amino acids, fatty acids, cholesterol, and glucose, this class also includes secondary metabolites such as lipids, alkaloids, glycosides, and natural polyphenols. Some chemicals, known as enzyme inhibitors, prevent enzymes from carrying out their typical job. They obstruct the function of critical enzymes, stopping the signaling process needed for the growth of cancer cells. By inhibiting these cellular signals, cancer may be stopped in its tracks and its capacity to spread. Small molecules may be applied as drugs to mute protein expressions in cancer cells [18–21].

This study focuses on a thorough analysis of the pharmacological effect of small molecules on genitourinary cancer and provides a method for effectively treating cancer.

## Pathological features

Genitourinary cancer is a distinct subtype of cancer that can invade any part of the genitourinary system, including the kidneys, testicles, prostate, and bladder [22, 23]. Early studies in epidemiology have identified a genetic component of cancer, and subsequent linkage research has uncovered a variety of rare syndromes, including cancers of the genitourinary system [24–26]. RCCs often present with visible or non-visible hematuria, flank mass, flank pain, or non-specific symptoms such as lethargy or weight loss, with approximately 50% of diagnosed cases detected incidentally on abdominal imaging. It may be the only cancer that can be treated surgically, even with metastatic foci [27]. A rise in the number of times a patient needs to urinate, the presence of blood in the urine, and painful or difficult urination are all symptoms of urological cancers, and there is a possibility that malignant growths are present if one has these symptoms [28].

On the other hand, tumor cells derived from testicular germ cells are often curable with chemotherapy, even in the presence of extensive metastases [29]. A number of disorders are linked to an increased risk of testicular cancer. Two of these conditions are Down syndrome and testicular dysgenesis syndrome [30]. The most common genitourinary cancer is prostate cancer. It is characterized by weakening of bone metastases, relatively slow behavior, and dependence on androgen receptor pathways, all of which can be addressed with extraordinary efficiency [31, 32]. Both the androgen receptor signaling pathway and testosterone are essential for the formation of prostate epithelium and prostate cancer cells [33, 34].

In comparison to Caucasian American males, African-American men have a greater mortality rate from prostate cancer, are diagnosed at a younger age, and have a higher risk of developing the disease [35]. Kidney cancer is an unusual "interstitial disease" linked to several metabolic, paraneoplastic, and hormonal problems that often accompany the metastatic stage [36, 37]. This type of cancer can undoubtedly form one of the most unexpected tumors. RCC is a type of cancer originating from cells located in the renal cortex. The renal pelvis is the birthplace of transitional cell carcinomas such as urothelial carcinomas. These malignancies are classified as urothelial carcinomas and are more likely subdivided into subtypes [38, 39].

Since the development of renal carcinoma has been observed to be linked with defects in the VEGF and mTOR pathways, drugs capable of blocking travel signals through these channels are an effective treatment for renal carcinoma [40]. It is the most common and successful treatment of testicular cancer with combination chemotherapy based on cisplatin-enhanced drugs, the benefits of which have led to a more favorable prognosis and outcome for people affected by the condition spreading to other parts of the body [41, 42]. This drug is a standard treatment for various visceral multi-organ system diseases. Unfortunately, bladder or urothelial cancer represents a class of neoplasms for which very little progress has been made in developing treatment options for metastatic disease [41, 43, 44]. Although many factors increase the probability of developing BC, a significant number of patients are diagnosed with the disease despite not having any evident exposures [45, 46]. Genetic or epigenetic changes may lead to modifications in tumor suppressor genes, growth factor receptors, and DNA repair genes. Identifying and characterizing molecular biomarkers and expression patterns associated with BC can aid in early detection and prediction of onset or relapse [47]. People diagnosed with BC usually display painless visible hematuria, a common symptom in many patients [48, 49].

## Therapeutic targets of small molecules in genitourinary cancers

**Fig. 1:**
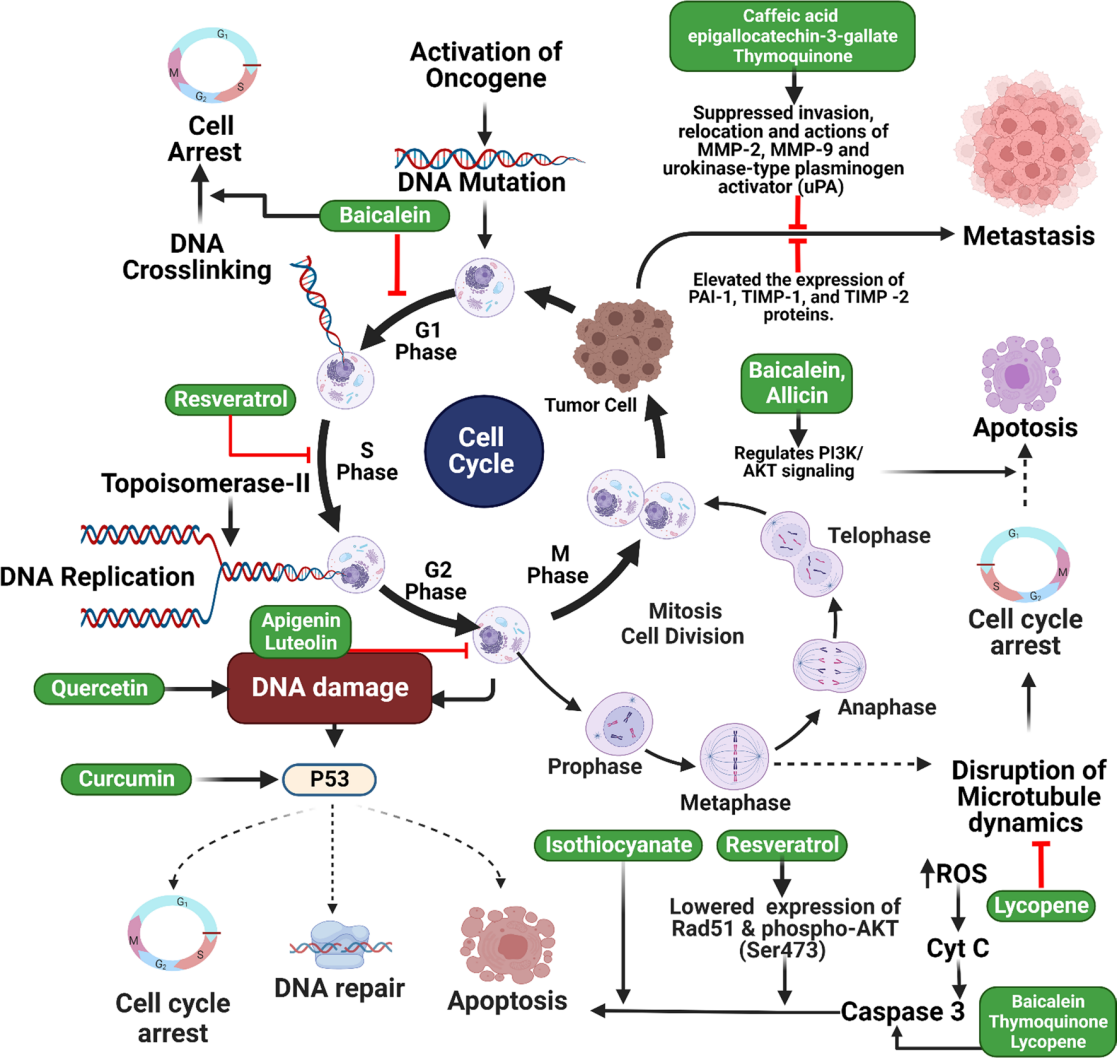
Illustration representing the site of action of different natural small molecules in common genitourinary cancer pathways

Phytochemicals derived from plants have shown promise in discovering new anticancer-effective and safe treatments [50, 51]. Cell cycle regulation has a considerable effect on the proliferation of tumor cells and their ability to metastasize and recurrence [52, 53]. Dysregulation of markers connected to cell-cycle regulation is the most widely researched molecular aberration in BC [54]. The inhibitory effect of baicalein on cell growth in T24 cells has been reported, whereby it induces a cell cycle arrest, specifically in the G1/S phase. The induction of apoptosis in Bacillus cells results from the activation of caspases 9 and 3 [55]. Resveratrol causes the advancement of LNCaP cells towards the S phase of the cell cycle when added to the culture medium of these cells. Because of the DNA synthesis-inhibiting properties of resveratrol, especially at doses more than 15 µM, it is difficult for the cell to continue developing in the S phase [56]. When it was discovered that practically all cancer-causing chemicals are also mutagenic, meaning they affect the DNA sequence, it became evident how significant DNA damage is in carcinogenesis [57]. However, the potential of quercetin to suppress the proliferation and colony formation of human BC cells has been observed through its ability to induce DNA damage [58]. Losing p53 activity alters cell growth, longevity, and resistance to cytotoxic drugs [59]. The administration of curcumin-induced apoptotic cell death in human bladder cancer cells, leading to their arrest in the G2/M phase of the cell cycle. The administration of curcumin decreased the levels of the anti-apoptotic proteins Bcl-2 and Survivin, accompanied by an increase in the levels of Bax and p53 [60]. In human BC cells, luteolin increased p21 expression and an arrest in the G2/M phase of the cell cycle [61]. Apigenin has been seen to induce the upregulation of Bax and Bad, leading to the activation of caspase-3 and poly (ADP-ribose) polymerase (PARP) (Fig. 1). Additionally, it has been found to block the PI3K/Akt pathway, resulting in the downregulation of anti-apoptotic proteins Bcl-2 and Bcl-x. Furthermore, apigenin has been shown to induce G2/M cell cycle arrest, specifically in T24 BC cells [62]. Activating caspases is an essential step in the process of apoptosis [63]. Caspase-3 facilitates cellular death and generates apoptotic bodies [64]. One of the most prominent roles of phosphatase and tensin homolog deleted on chromosome 10 (PTEN) is its activity as a negative modulator of the PI3K/Akt/mTOR pathway, a critical signaling system involved in the growth of cancer cells [65, 66]. Allicin can inhibit the biological activity of BC cells by efficiently reducing the expression of miR-26b-5p/PTEN [67]. (−)-epigallocatechin-3-gallate causes substantial discrepancies in the mRNA gene expression of the growth signaling pathway activation in normal and tumorigenic human BC cell lines (Fig. 1) [68].

## Role of small molecules in genitourinary cancers

### Bladder cancer

BC is the fourth most common cancer in the West and ninth in women [69]. Researchers have gone through many wet-lab experiments to explore the role of small molecules in this regard. A study investigated the effects of resveratrol, a polyphenolic compound found in strawberries and red wine, on bladder cancer cell (BCC) proliferation and apoptosis. The study found a dose-dependent increase in cytotoxicity and apoptosis when resveratrol was added to 5637 and T24 cell lines. The protein levels of miR-21, phospho-act, and Bcl-2 dropped in response to resveratrol supplementation. Low levels of miR-21 control expression, reducing phospho-act and BCL-2 levels. Insulin-like growth factor-1 attenuated the influence of MIR-21 inhibitor Bcl2 on apoptosis in T24 and 5637 cells. Resveratrol's effects on cell death, such as inhibition of Akt activity, reduced Bcl2 expression and increased miR-21 expression, counteracted apoptosis [70]. Finally, resveratrol induced significant S phase arrest and apoptosis, as well as decreased STAT3 phosphorylation, nuclear translocation, and transcription, as well as downregulation of STAT3 downstream genes (including C-Myc, CyclinD1, VEGF, and Survivin) and nuclear translocation of P53 and CIRT1. Tyrphostin AG490, an inhibitor of JAK2, was used to prove without a shadow of a doubt that the STAT3 signal is crucial to cell proliferation after being tested on EJ cells. The findings of the naked mouse orthotopic xenograft model show that resveratrol installation is efficient and safe since it inhibits tumor development after transplantation without influencing normal urothelium, individual apoptosis, or STAT3 inactivation. This suggests resveratrol may be an effective and safe treatment for bladder transitional cell cancers postoperatively [71].

Curcumin has received a lot of interest as a promising anti-cancer medicine owing to its ability to alter many pathways and genes. Research has demonstrated that the levels of Sp4, Sp3, and Sp1 are reduced in a proteasome-dependent manner in the curcumin-treated KU7 and 253JB-V cells. RNA interference inhibits curcumin-dependent NF-kB binding genes, including BCL-2, survival, and cyclin, by inhibiting short-resistance RNAs targeting Sp4, Sp3, and Sp1. It contributed to the deficiency in D1 and SP proteins. In a study using xenografted KU7 cells in the bladder of primitive naked rats, curcumin significantly reduced tumor growth. After that, Sp4, Sp3, and Sp1 tumor protein levels were lowered [72]. Pro-survival Bcl-2 and the anti-apoptotic Bax proteins are under the therapeutic control of curcumin, which is associated with an upregulation of Bax and p53 expression. Curcumin had a more potent inhibitory effect than cisplatin, while pre-treatment of T24 cells with catalase did not affect them. Clonal testing has established that large doses of curcumin administered rapidly are lethal to BCCs. Curcumin inhibits and halts the growth of BC by triggering apoptosis in vivo. According to this data, curcumin shows promise as a chemo-preventive and chemotherapeutic drug for bladder cancer [60]. So far, quercetin has not been approved for clinical trials despite promising preclinical results in preventing many cancer types, including liver, breast, nasopharyngeal, and prostate. In vitro, MTT, and collagenic acid assays tested rats for bladder cancer cell-resistant sensitivity. Afterward, AMPK pathways were analyzed using Western blotting to see how 4E-BP1 and S6K fit into the picture, with the quercetin inhibiting cell migration and inducing apoptosis [73]. After transurethral resection for papillary urothelial bladder cancer, Quercetin has shown promise as both a chemotherapeutic and a chemotherapeutic agent. This is because research on quercetin has revealed that it damages DNA, which inhibits the growth and spread of human BCCs. Human BCCs may multiply and spread, although some research suggests Quercetin might slow that process [58]. Several cancers have been demonstrated to be effectively stunted by apigenin. Apigenin treatment reduces transplanting and BCC attack risk by inhibiting cell invasion and migration, potentially involved in the Bcl-2 family action mechanism and PI3K/Akt pathway. It induces apoptosis through increased PARP cleavage and caspase-3 activity [62].

Apigenin significantly reduced MAPK activation, phosphorylation, and HBSM cell motility. Apigenin inhibits actin polymerization, which is required for cellular motility and muscle contraction. As the findings show, it appears to prevent MAPK activation and cell transfer. It may work by blocking the signal from MEKK1 to MAPKs [74]. Apoptosis activation is the fundamental mechanism that makes isothiocyanates a promising anti-cancer medication. Specifically, isothiocyanates cause BCL-2 phosphorylation, cause Bak translocation to mitochondria, and block BCL-XL's ability to interact with Bak and Bax at mitochondrial membranes. Based on these findings, some Bcl-2 family members contribute to isothiocyanate-induced mitochondrial damage [75]. As shown in BC xenografts, sulforaphane and erucin inhibit histone distillation activity in human BCCs, ranging from superficial to invasive. This suggests studying isothiocyanates' role in inhibiting histone H1 phosphorylation as a new research direction [76]. A study investigated the flavonoid baicalein found in the roots of *Scutellaria baicalensis* Georgi. Flow cytometry and nuclear structure analysis showed that bicarbonate therapy significantly slowed cell development when concentration was increased. This obstruction was linked to increased apoptosis, and the Poly (ADP-Ribose) Polymerase during the process of creating baicalein 5637 cells degraded by proteolysis, leading to a decrease in the activation of apoptosis proteins Caspase-9 and -3 in the immunological family. Regulated inhibitors of apoptosis protein (IAP) family members like CIAP-2 and CIAP-1 effectively reduced cell death through apoptosis. Basil stimulated Caspase-8, improved bead pruning, and raised death receptors. Pan-caspase inhibitors reversed baseline-induced apoptosis, suggesting caspase activity as the route. Baicalein's action on MMP and caspase activation could be mitigated by pre-treatment with basil's antioxidant N-acetyl-L-cysteine, which stimulates the generation of reactive oxygen species (ROS) [77]. In T24 cells, baicalein inhibited cell growth by causing a cell cycle arrest in the G1/S phase. Caspases 9 and 3 are activated, and apoptosis is induced in Bacillus cells. The ratio of Bcl-2 to Bax rises due to the potency of baicalein. Bcl-2 is regulated, and the expression of Bax is increased when Akt phosphorylation is inhibited [55]. The primary active constituent of black seed oil, thymoquinone, has been demonstrated to have anti-cancer activity. Thymoquinone has potent cytotoxic effects on BCCs.

The anti-cancer properties of thymoquinone were found to be linked with mitochondrial dysfunctions and endoplasmic reticulum stress-related proteins. Apoptotic effects of thymoquinone can be partially mitigated through the inhibition of CHOP using pan-caspase inhibitors such as Z-VAD-fmk or endoplasmic reticulum stress inhibitors like 4-PBA or shRNA. The objective can be achieved by increasing the production of Bcl-2, preventing cytochrome C release, and preventing the relocation of Bax from the mitochondria to the cytoplasm [78]. Thymoquinone suppressed the expression of genes such as MMP7, MYC, Axin-2, MET, and CyclinD1, which are known to be regulated by β-catenin. These genes are crucial for epithelial-mesenchymal transition (EMT) and cancer development. Thymoquinone may inhibit the activation of the Wnt/β-catenin signaling pathway. Additionally, it has been demonstrated that thymoquinone may inhibit the growth of xenografts and reduce the number of metastatic tumor foci that establish themselves in the lungs [79].

The bioactive chemical in propolis, caffeic acid phenethyl ester, is effective against cancer. In vitro studies have linked GDF15 expression in epithelial cells to a decreased risk of neoplasia. Caffeic acid phenethyl ester improved the expression of maspin and NDRG1. Decreasing the proliferation and invasion of BCCs in vitro of the GDF15 was necessary for the observed effect. Results from xenograft animal studies indicate that caffeic acid phenethyl ester may reduce tumor development in living organisms. Caffeic acid acted independently of AMPKα1 and AMPKα2 to alter GDF15 expression by increasing phenethyl ester ERK, JNK, and p38 activation. Pre-treatment with ERK, JNK, or p38 inhibitors mitigated NDRG1, GDF15, or maspin activation by caffeic acid phenethyl ester. AMPKα1/2 knockdown reduced caffeic acid phenethyl ester-induced GDF15 expression and bladder cancer cell proliferation [80]. Garlic's (*Allium sativum* L.) defensive molecule, allicin (Diallyl disulfide), has a wide variety of biological effects [81]. Reductions in miR-26b-5p were substantial and dose-dependent, and there was a substantial upregulation of PI3K, AKT, and PTEN proteins and a corresponding downregulation of PI3K, AKT, and PTEN proteins [67]. Luteolin, a natural flavonoid, exhibits anti-cancer properties in cancers other than bladder cancer. Luteolin causes cell cycle arrest in the G2/M phase and cell death.

Moreover, luteolin activates transient receptor potential channel 1 (TRX1), inhibiting intracellular ROS production. In a mouse model of BC31 rat bladder cancer implanted subcutaneously and administered luteolin orally, tumor volume decreased significantly. As a comparison, it was tested on a group of animals used as a control. According to immunohistochemistry results, p21 was overexpressed, and p38 was suppressed in the luteolin treatment group. In models of in vivo *N*-butyl-*N*-(4-hydroxybutyl) nitrosamine (BBN) producing rat bladder cancer, oral treatment with luteolin led to a trend toward a lower risk of bladder tumors. The Ki67 index and p38 expression dropped significantly; due to this, the quantity of luteolin-3′-o-glucuronide in urine and plasma is highly connected to cell proliferation and repression of mTOR signals, according to the most important studies on luteolin metabolism. The plasma concentration of luteolin-3′-glucuronide has also been linked to a dramatic decline in the incidence of squamous cell carcinoma and BC [61]. *Camellia sinensis* L., often known as KTZE green tea, is a member of the family Theaceae and contains a polyphenolic compound called epigallocatechin-3-gallate. Several catechins are connected to this polyphenolic component and are responsible for green tea's health advantages [82]. Microarray studies demonstrated differential mRNA gene expression in response to epigallocatechin-3-gallate stimulation of growth signaling pathways in normal and tumorigenic human bladder cell lines. These findings suggest that polyphenols in green tea may alleviate bladder disorders (Table 1) [68].

**Table 1:** Experimental evidence on the use of natural small molecules in genitourinary cancers

Cancer types	Compounds	Study model	Dose	Results	Mechanism of action	Refs.
Bladder cancer	Resveratrol	In vitro (5637 and T24 cells)	10, 30 and 50 μmol/L	Cell apoptosis	Control of the Akt/Bcl2 signaling pathway	[70]
		In vivo and in vitro (mice and human TCC EJ cell lines)	150 µM or 200 µM	Cell apoptosis	Significant S phase arrest and apoptosis decreased STAT3 phosphorylation, nuclear translocation, transcription, and downstream gene expression, including c-Myc, survivin, VEGF, and cyclinD1	[71]
	Curcumin	In vitro (Sp3, Sp4, and Sp1 in KU7 and 253JB-V cell lines)	10–25 μmol/L	Anti-tumor effect	Arrest NF-kB dependent genes including cyclin D1, bcl-2 and surviving	[72]
		In vitro and in vivo (T-24 UMUC2 and EJ cell line and Wistar rats)	40 μmol/L	Apoptosis	Downregulation of Bcl-2	[60]
	Quercetin	In vitro (T24, UMUC3, and MB49 cell lines)	0, 5, 10, 20, 40, and 80 µM	Induction of apoptosis	Initiation of AMPK pathway	[73]
		In vitro (T24 cell line)	1 and 50 µM	Cell proliferation inhibited	Induction of DNA damage	[58]
	Apigenin	In vitro (T24 cell line)	10–80 Μm	Cell proliferation suppressed	Cell cycle is arrested in G2/M, and caspase-3 and PARP cleavage are up-regulated	[62]
		In vitro (Human bladder smooth muscle cells)	50 μM	Cell migration inhibited	Restricts the initiation of MAPKs	[74]
	Isothiocyanate	In vitro (UM-UC-3 cell line)	7.5, 15, and 30 μmol/L	Induction of apoptosis	Resulting in the phosphorylation of Bcl-2, the induction of the translocation of Bak into the mitochondria	[75]
		In vitro (RT4, J82 and UMUC-2)	20 μM	Reduced cancer cell progression	Proteomic changes in histones following exposure to isothiocyanates	[76]
	Baicalein	In vitro (5637 cell line)	150 μM and 250 μM	Induction of apoptosis	cIAP-2 and cIAP-21downregulation, caspase-9 and -3 activation, Bax decrease, and Bcl-2 initiation	[77]
		In vitro (T24 cell line)	80–120 μmol/L	Inhibited cell growth	G1/S cell cycle arrest results in reduced mitochondrial transmembrane potential damage. In addition, Akt phosphorylation is inhibited, Bax expression is elevated, and Bcl-2 expression is decreased	[55]
	Thymoquinone	In vitro (T24 and 253j bladder cancer cell)	20–160 μmol/L	Cytotoxic effect	Alterations in the cytochrome C, Bax, Bcl-2, and endoplasmic reticulum are stress-related (Caspase-12, CHOP, and GRP78)	[78]
		In vivo and in vitro (Mice, and T24 and 253j cell lines)	10–120 μmol/L	Inhibits metastasis and cell growth	inhibiting the production of β-catenin target genes	[79]
	Caffeic acid	In vitro and in vivo (HT1376, RT-4, T24, TSGH-8301 cell lines and BALB/cAnN-Foxn1)	0–30 μM	Anti-tumor effect	Modulated GDF15 expression	[80]
	Allicin	In vitro (5637 and T24 cell lines)	–	Induction of apoptosis	miR-26b-5p was dramatically downregulated. The proteins AKT, PTEN, and PI3K were highly up-regulated, whereas tgf, PTEN, and PI3K were dramatically down-regulated	[67]
	Luteolin	In vitro and in vivo (T24 cells and KSN nude mice)	10–25 µmol/L	Cancer growth suppression	G2/M cell cycle arrest	[61]
	(-)-epigallocatechin-3-gallate	In vitro (UROtsa, Sw780, TCCSUP)	0–80 μg/mL	Apoptosis induction	PI3K/Akt pathway activation	[68]
Renal cancer	Resveratrol	In vitro (ACHN and 786-O)	–	Cell proliferation inhibited and apoptosis induced	Reduced expression of Shh pathway-associated proteins	[83]
		In vitro (786–0 cells)	0, 10, 20 and 40 µmol/l	Anti-tumor effect	VEGF gene expression inhibition	[84]
	Curcumin	In vitro (RCC-949)	0–100 µM	Anti-cancer effect	Bcl-2 expression initiated, Bax expression reduced, and PI3K/AKT signaling pathway activation decreased	[85]
		In vitro (786-O cell)	0, 6.25, 12.5, 25, 50 µmol/L	Induction of apoptosis	The expression levels of MTOR, MMP9, MMP2, and p-MTOR proteins rapidly reduced	[86]
	Apigenin	In vitro and in vivo (786–0, ACHN, and Caki-1 cells, nude BALB/c mice)	30 mg/kg	Cell proliferation reduced	Induction of G2/M phase cell cycle arrest	[87]
		In vitro (ACHN, Caki‑1, and NC65)	1–100 µM	Cell growth inhibited	cell cycle arrest at G2/M	[88]
	Isothiocyanate	In vitro (GRC-1 cell)	0, 7.5, 15, and 30 μM	Induction of apoptosis	Increased Bax and decreased Bcl-2 protein level	[89]
	Thymoquinone	In vitro (Caki-1, Caki-2, A498)	0, 1, 2, 5, or 10 μM	Exerted anti-cancer effect	Inhibited hypoxia-inducible factor-1α	[90]
		In vivo and in vitro (Mice and Caki-1)	-	Initiated apoptosis	JAK2/STAT3 signaling pathway suppression	[91]
	Lycopene	In vivo (Female Eker rats)	100 and 200 mg/kg	Tumor growth decreased	Regulation of mTOR, phosphor-S6, and EGFR	[92]
	Caffeic acid	In vivo and in vitro (BALB/c mice and caki-1 cell)	5 mg/kg	Reduce tumor angiogenesis	Inhibited the activity of STAT3 and HIF-1α	[93]
		In vitro (RCC-9863 Cells)	0.016, 0.05, and 0.1 mg/mL	Inhibited cancer cell growth	The protein level of HIF-1α was observed to have undergone a significant decrease	[94]
Prostate cancer	Resveratrol	In vitro and in vivo (PC-3 M-MM2, DU145 and nude mice)	5–100 µM	Anti-tumor action	Increased expression of PCDC4, maspin, and reduced MiR-21 expression	[95]
		In vitro (DU145 and LNCaP)	5–10 μm	Induction of apoptosis and anti-proliferative action	Inhibited DNA synthesis	[56]
	Curcumin	In vivo and in vitro (Mice and DU145)	10 μm	prevented cancer cell progression	MMP-9 and MMP-2 decreased	[96]
		In vitro (LNCaP and PC-3 cells)	40 and 30 μm	Reduced cancer cell progression	The observed effect is a decrease in the transactivation and expression of the androgen receptor	[97]
	Quercetin	In vitro (PC-3 cell line)	50 and 100 μM	Inhibited metastasis	Downregulated uPA, uPAR, and EGF, EGF-R mRNA expressions inhibited β-catenin, NF-kB, N-Ras, p-EGF-R, Raf-1, c.Fos c.Jun and p–c.Jun protein expressions	[98]
		In vivo and in vitro (Mice, and HEK293, CWR22Rv1 cell lines)	0, 10, or 20 μmol/L	Ameliorated prostate cancer resistance	Reduces hnRNPA1 and AR-V7 expression	[99]
	Apigenin	In vitro and in vivo (LNCaP, PC-3 cell lines and mice)	20 μg	Suppressed cancer cell progression	Regulation of PI3K/Akt/FoxO-signaling pathway	[100]
		In vivo and in vitro (Mice, and DU145, PC-3 cell lines)	20 μM	Induction of apoptosis	Protein levels of c-IAP2, XIAP, c-IAP1, and survivin decreased	[101]
	Isothiocyanates	In vitro and in vivo (PC-3 and LNCaP cell lines and mice)	5 μmol/L	Induction of apoptosis	Suppressed Akt, mTOR	[102]
		In vitro (PC-3 and LNCaP)	5 μm or 10 μm	Induction of apoptosis	Reactive oxygen species production leads to cancer cell death	[103]
	Thymoquinone	In vitro (DU145 and PC3 cells)	10.0 µM	Inhibited metastatic property	Downregulated TGF-β/Smad2/3 signaling pathway	[104]
		In vitro (DU-145, LNCaP, and PC-3 cell lines)	10.18, 12.40, and 16.78 µM	Suppressed tumor growth	Downregulated p-Akt, NF-kB signaling pathway	[105]
	Caffeic acid	In vivo and in vitro (Mice, and PC-3, DU-145, LNCaP cell lines)	10 mg/kg	Suppressed cancer cell growth	Inhibited PI3K/Akt signaling pathway	[106]
	Epigallocatechin gallate	In vitro (PC-3ML)	0–40 µg/mL	Cancer cell growth is inhibited	Inhibits prolyl hydroxylation of HIF-1α	[107]
Testicular cancer	Curcumin	In vitro (NTERA-2 and F9 cell lines)	0–15 µmol/L	Inhibited proliferation and induced apoptosis	Inhibition of AP-2γ-mediated downstream cell survival signaling pathways	[108]

### Renal cancer

Renal cell carcinoma (RCC) is a common adult-onset disease, affecting 3% of malignancies and 85% of kidney tumors in adults. Researchers are studying the role of small molecules in RCC, finding that resveratrol treatment reduces cell sphere size, quantity, and expression of the Shh pathway and cancer stem cell marker. At the same time, purmorphamine increases the Sonic Hedgehog pathway [83]. Resveratrol, when administered at different concentrations to 786–0 cells, suppressed the VEGF gene, with the dose being proportional to both concentration and treatment duration, demonstrating its anti-cancer activity [84].

Curcumin inhibits cell survival and growth, whereas it has a stimulating effect on cell death. This effect was linked to increased Bcl-2 and decreased Bax expression. Cells treated with curcumin enter a heightened state of cell cycle arrest, possibly due to reduced cyclin B1 expression, as seen in cells treated with curcumin. The presence of curcumin makes PI3K / AKT signaling pathways less active [85]. The expression of MMP9, p-MTOR, MTOR, and MMP2 proteins, as well as the cells at stage S, increases as the curcumin concentration increases.

Conversely, the number of cells in the G2 / M and G1 stages and the cell death rate continue to increase. Depending on concentration and time, 786-O cell proliferation in each treatment group decreased. Cell migration and the percentage of transmembrane cells decreased significantly as the resistance rate increased [86]. Apigenin inhibited 786–0, ACHN, and Cake-1 RCC cell growth. In ACHN cells, a comet test revealed that apigenin caused exceptionally high DNA damage levels. Apigenin-induced apoptosis depends on p53 using small interfering RNA (siRNA), which was used to induce p53. An ACHN cell xenograft rat model was used to verify that apigenin inhibits cancer cell proliferation. Apigenin therapy reduces tumor size growth in vivo, and immunohistochemistry labeling has shown low Ki-67 indicators in tumors made from apigenin-treated rats [87].

Apigenin was added to human RCC NC65, Cake-1, and ACHN at concentrations ranging from 1 to 100 μM for 24 h, inhibiting cell growth proportionally to the amount. Cytostatic effects were observed even when the treatment duration was reduced to 3 h. Apigenin reduced cyclin D3, A, B1, and E levels in G2/M phase cell cycle arrest and RCC cells, resulting in the inhibitory growth of density-dependent cells, also observed in early RCC cells [88]. In a dose-dependent manner, isothiocyanate decreases the proliferation of GRC-1 cells and promotes cell death. In addition to reducing the BCL-2 gene expression, it also increases Bax. In general, increasing the concentration of isothiocyanate minimizes the ratio of Bcl-2/Bax [89]. Thymoquinone, a new HIF-1α inhibitor, has been discovered by screening 502 natural chemicals at the luciferase assay-Based Chemical Library. Thymoquinone reduces HIF-1α protein levels in renal cancer cells but does not affect HIF-1α protein levels when combined with the proteasome inhibitor MG132. Thymoquinone increases the rate of degradation of HIF-1α protein by inhibiting the interaction between HIF-1α and HSP90. It also inhibits downstream gene replication of HIF-1α, affecting its transcriptional activity [90]. When high doses of thymoquinone were applied to Cake-1 cells, the expression of p53 and Bax was increased, while the expression of anti-apoptotic genes was regulated.

Moreover, thymoquinone inhibited the JAK2/STAT3 pathway, resulting in a decrease in the production of oncoproteins that promote cell survival. It interacts with BCL family members to promote apoptosis in Cake-1 cells. In nude rats, thymoquinone inhibited Cake-1 cell-produced tumor xenografts [91]. Dietary lycopene reduces renal cell tumor development in sensitive TSC2 mutant-acre rat models [92]. Caffeic acid has been shown to reduce the activity of STAT3, decreasing HIF-1α activity dramatically; studies have discovered this.

Consequently, the gradual blocking of STAT3 and HIF-1α caused VEGF downgrades due to their placement in the VEGF promoter [93]. Allicin significantly reduces HIF-1 protein levels, decreasing both BCL-2 and VEGF expression. In addition to these, it is clear that allicin promotes apoptotic cell death. After treatment with allicin, RCC-9863 slows down cell colonization and motility. Further studies have shown that an additional expression of HIF-1α may partially counteract the effects of allicin (Table 1) [94].

### Prostate cancer

Resveratrol has been found to decrease the production of microRNAs associated with prostate cancers, reducing cellular viability, migration, and invasiveness. It may also increase the expression of PDCD4 and maspin, both genes regulated by miR-21. However, siRNA directed against PDCD4 in prostate cancer cells (PCCs) diminished resveratrol's effects. Both resveratrol and LY294002 successfully reduced phospho-Akt levels in PC-3M-MM2 cell lines, with LY294002 reducing miR-21 levels and increasing PDCD4 expression simultaneously. Oral treatment with resveratrol slowed tumor growth and reduced the frequency of metastatic lung lesions. These anti-tumor and anti-metastatic effects were connected with decreased levels of pAkt, miR-21, and greater levels of PDCD4. The anti-tumor activities of resveratrol in PCCs derived from LNCaP and DU145 were found to be equivalent, with the decrease of PDCD4 and Akt, while miR-21 did not influence them [95]. A study found that resveratrol inhibited DNA synthesis, with a more prominent effect when the compound was increased. However, resveratrol had a twofold effect on DNA synthesis when the treatment was extended to a full day. At concentrations between 5 and 10 µm, DNA synthesis increased by 2–3, while at concentrations less than 15 µm, it prevented it. Only androgen-independent LNCaP PCCs, andro-gen-independent DU145 PCCs, and NIH3T3 fibroblast cells showed increased DNA synthesis. There was a correlation between an increase in the percentage of LNCaP cells in the S phase of the cell cycle and a decrease in nuclear p21Cipl and p27Kip1 levels, which co-occurred with the increase in DNA synthesis by resveratrol. Additionally, there was a significant increase in nuclear Cdk2 activity associated with cyclin E and A. This conclusion supports the observation that LNCaP cells progressed into the S phase of the cell cycle [56].

**Fig. 2:**
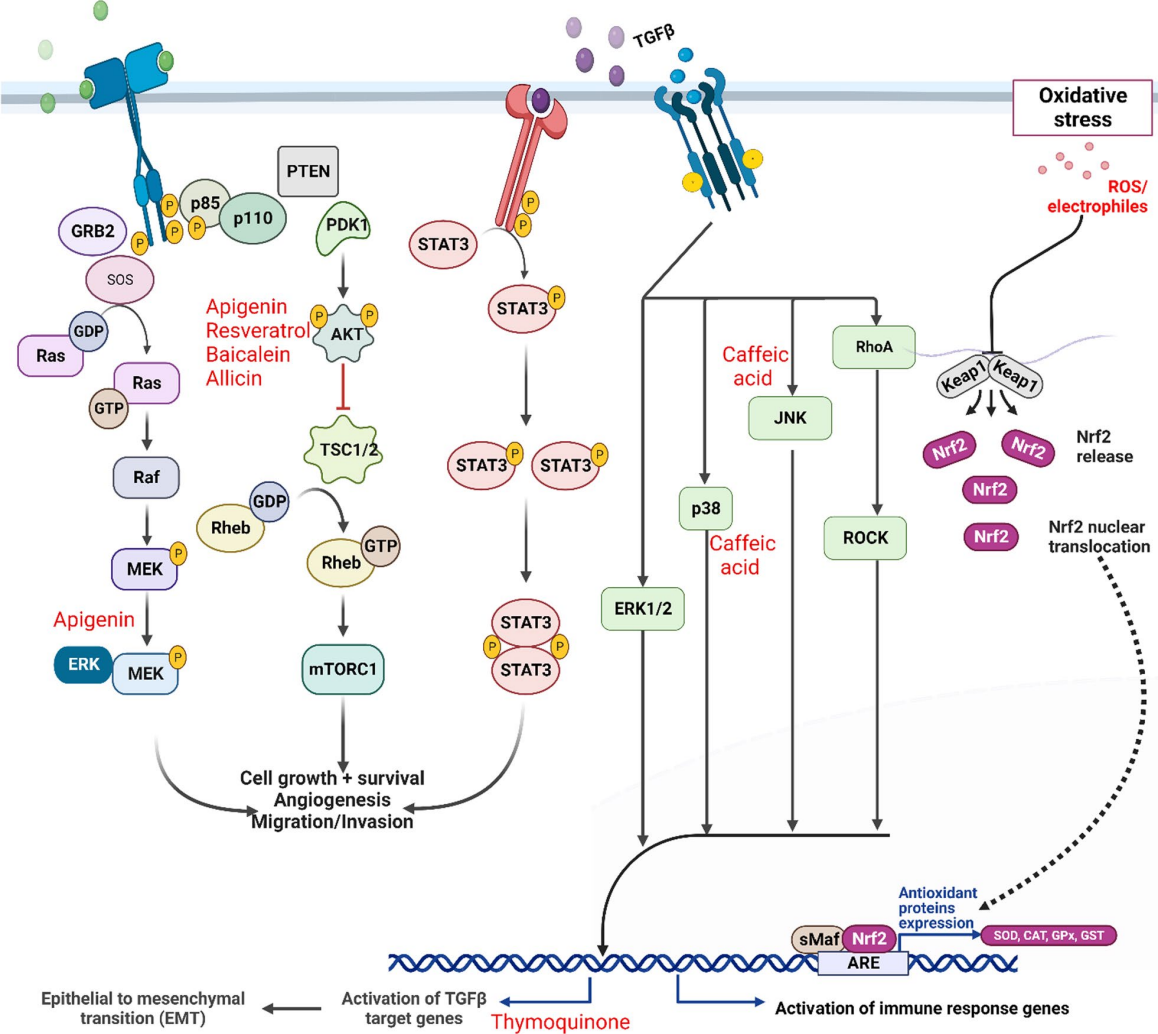
Exploring the roles of some small molecules on signaling pathways in genitourinary cancers

Curcumin treatment not only reduced the synthesis of MMP-9 and MMP-2, but it also hindered the invasive capability of the cells when examined in vitro. It has been shown that curcumin may be responsible for a considerable reduction in tumor volume and MMP-9 and MMP-2 activity in the region where the tumors are situated. Compared to the therapy that served as the control, curcumin treatment decreased the number of metastatic nodules seen in vivo, which was statistically significant. Curcumin shows promise as a prospective treatment for preventing cancer formation or, at the very least, the early phase of metastasis. This promise is shown in prostate cancer [96]. The potential of curcumin to downregulate transactivation and expression makes it possible for it to suppress the function of several transcription factors, including the androgen receptor, activator protein-1 (AP-1), NF-kB, and cAMP. Curcumin may have a therapeutic effect on PCCs by inhibiting the activity of the androgen receptor and other cofactors associated with the androgen receptor [97]. When PC-3 cells are treated with quercetin, the capacity of the cells to invade and migrate into the surrounding tissue is significantly diminished. Quercetin can inhibit mRNA expression for uPA and uPAR, in addition to EGF and EGF-R. The protein expressions of cell survival factor catenin, nuclear factor NF-kB, and proliferative signaling molecules including p-EGF-R, Raf-1, N-Ras, c.Jun, c.Fos, and p–c.Jun are all suppressed by quercetin. In addition, quercetin can inhibit the formation of the protein p–c.Jun. On the other hand, quercetin increased the quantity of p38 MAPK protein (Fig. 2) [98]. Due to quercetin's ability to block the formation of hnRNPA1, the production of AR-V7 is also reduced. Quercetin's capacity to decrease AR-V7 activity may make it possible for enzalutamide-resistant PCCs to become susceptible to treatment with enzalutamide once again [99].

Apigenin treatment reduced photo phosphorylase of Akt (Ser473) and FoxO3a (Ser253) in rats, consistent with apigenin's greater atomic content. Additionally, 14-3-3 apigenin binding was reduced as a consequence of the treatment. These activities cause a decrease in the rate of cell proliferation, as assessed by the proteins Ki-67 and cyclin D1, and a rise in the levels of the FoxO-reactive protein BIM and p27/Kip1. Following treatment with apigenin showed the same findings in LNCaP and PC-3 cells, which supplemented the findings in vivo. Convulsions occurred during the G0/G1 stage of the cell cycle due to treatment with apigenin at concentrations of 10 and 20 µM, which caused FoxO3a binding to P27/Kip1 to increase significantly. This probe impacted cells comparable to that produced by the PI3K/Akt inhibitor LY294002 [100]. PC-3 and DU145 cell lines treated with apigenin showed a reduction in XIAP, c-IAP1, and c-IAP2 protein levels and survival. Apigenin treatment also increased cell viability and apigenin injection led to a considerable reduction in cell function as well as the induction of apoptosis, which was followed by a time-dependent rise in cytochrome *C*. Apigenin has been demonstrated to have these effects, which resulted in a reduction in Bcl-xL and Bcl-2 while an increase in the active form of Bax protein is seen. Apigenin was responsible for the development of Bax, which happened owing to the separation of Bax from Ku70, which is required for Bax to perform its apoptotic function. This was caused by the isolation of Bax from Ku70. Apigenin treatment inhibits class I histone dysentery and generates HDAC1 protein, both of which raise the level of acetylation of Ku70 in cancer cells and detach Bax, which ultimately leads to the death of cancer cells. In addition, apigenin causes a considerable reduction in HDAC1 uptake among XIAP promoters, suggesting that histone deformation XIAP may be a critical step in controlling downtime [101]. Both the ectopic expression of the active structural act and the excessive expression of the mTOR-positive regulator Rheb could only partly or partially reverse the processing and placement of LC3. Phenytoin reduces mammalian targets of dynamic phosphorylation and rheumatoid arthritis (mTOR) under the Isothiocyanate Treatment Akt, which happened despite phenytoin's role in autophagy regulation being mediated by various stimuli. In the presence of pharmacological autophagy inhibitors, the cells' phenethyl isothiocyanate-mediated apoptotic DNA fragmentation was considerably decreased (3-methyl adenine). PC-3 and LNCaP cells, which Atg5-specific short-interfering RNA had transiently translocated, were strongly protected against phenytoin isothiocyanate-mediated autophagy and apoptotic DNA fragmentation [102]. Phenethyl isothiocyanate prevents the oxidative phosphorylation process (OXPHOS) from causing cell death by preventing the generation of ROS. Exposure to pharmacological concentrations of phenethyl isothiocyanate produces ROS, inhibiting complex III activity and decreasing OXPHOS ATPs. However, the human prostate epithelial cell (PrEC) line showed no effects. Cyclosporine A did not alter ROS generation induced by PEITC treatment. Rho-0 variants of PC-3 and LNCaP cells show increased resistance to ROS, apoptotic DNA fragmentation, and possible mitochondria collapse. Treatment with phenethyl isothiocyanate activates the back in wild-type LNCAP and PC-3 cells but not the Rho-0 variant. RAC interference with Bax and Bak provides better protection against apoptosis from phenethyl isothiocyanate [103]. Thymoquinone has been found to prevent metastatic illness progression in PC-3 and DU145 cells by blocking EMT by reducing vimentin and elastin appearance and increasing E-cadherin expression. It may also connect signaling pathways related to TGF-β with EMT. Treatment with thymoquinone in these cell lines resulted in significant decreases in Smad3, TGF-β, and Smad2 expression levels. This decreased signaling pathway regulation, which TGF may partially reverse, may limit prostate cancer metastasis and EMT [104]. Thymoquinone effectively limited the proliferation rate of DU-145, LNCaP, and PC-3 cells, with GI50 values of 10.18, 12.40, and 16.78 M, respectively. DU-145 displayed higher expression levels of IL-7R and IL-7 in all PC types. IL-7 boosts metastatic incidence in DU-145 cells, inhibiting trans endothelial migration, cell proliferation, and cell invasion. Thymoquinone also inhibits p-Akt and NF-βB activity in DU-145 cells, leading to a dose-dependent reduction in MMP-7 and MMP-3 levels [105]. Caffeic acid phenethyl ester inhibits tumor development in PCCs (Table 1) [106]. The injection of epigallocatechin gallate under normoxic circumstances results in a dose-dependent rise in HIF-1 protein levels and HIF-1-intermediate transcription. However, when prostate cancer cells were treated concurrently with epigallocatechin gallate and iron-containing ions, only the treatment eliminated increased HIF-1α-mediated transcription. This was the case even though I gave both treatments simultaneously. Based on these findings, epigallocatechin gallate may serve as a chelator for iron-containing ions. In addition, epigallocatechin gallate inhibits a process known as the propyl hydroxylation of HIF-1α, which prevents this protein from interacting with PVHL [107].

### Testicular cancer

**Fig. 3:**
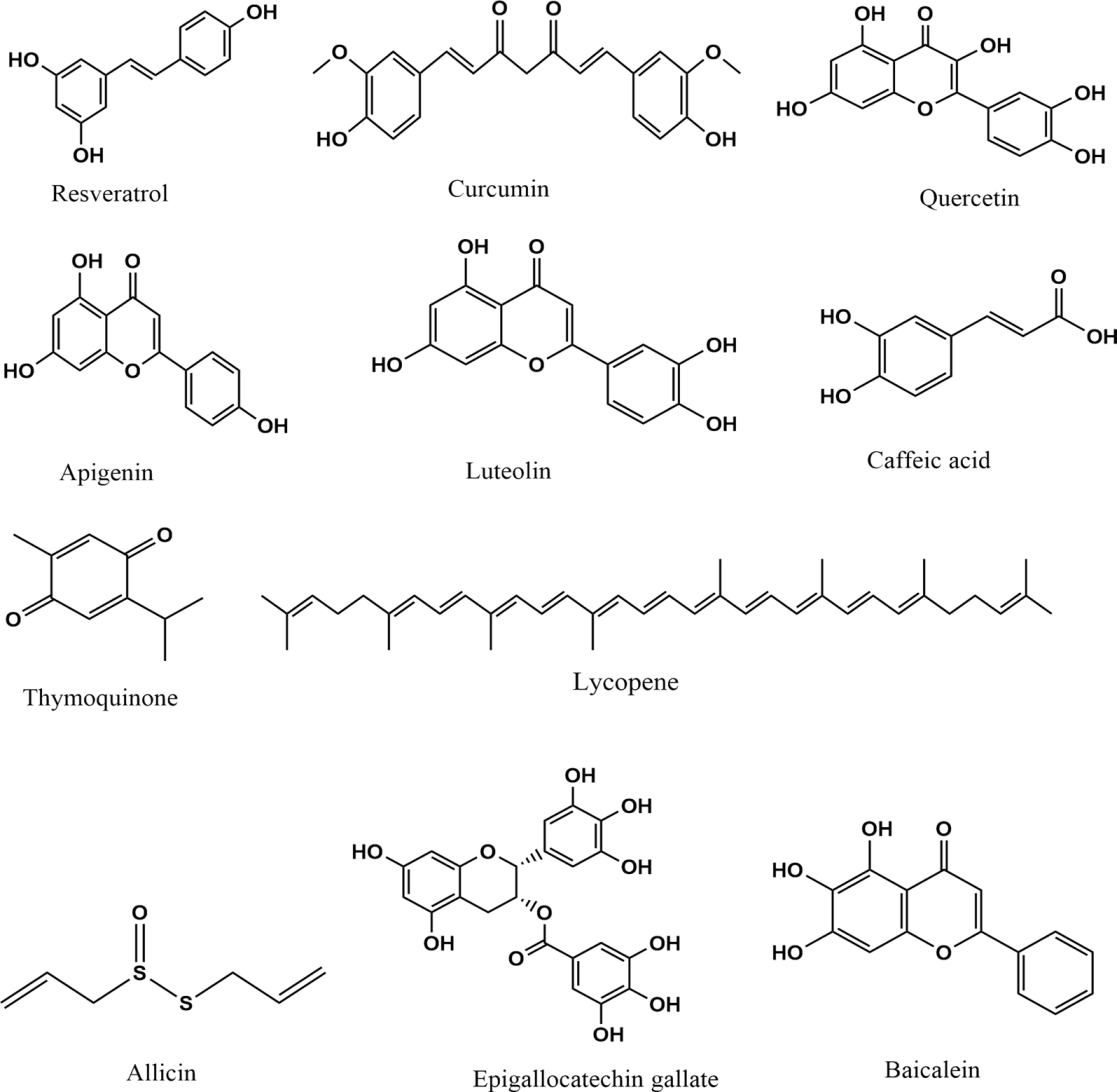
Natural small molecules effective in genitourinary cancers

Curcumin (Fig. 3), in a manner that was dependent on both dose and length of exposure, inhibited NTERA-2 cells from proliferation. NTERA-2 and F9 cells showed significant colony formation inhibition after curcumin treatment. Curcumin reduces FasL expression and the ratio of Bax to Bcl-2, leading to NTERA-2 cell death. This was done by curcumin's ability to activate caspase-3, -9, and -8 in a dose-dependent way, leading to the termination of NTERA-2 cells. In NTera-2 cells, curcumin decreased, dose-dependently, the expression of AP transcription factor AP-2γ, which is responsible for gene expression. Curcumin suppressed ErbB2 synthesis in NTERA-2 cells and decreased Akt and ERK phosphorylation in these cells (Table 1) [109].

The small molecules effective in genitourinary cancers are shown below.

## Synergistic effects

Among all cancers, genitourinary cancers are more prevalent in men [110, 111]. Kidney, prostate, and bladder cancers are a few urethral malignancies [112]. Even when the disease progresses to the first therapy, the 5-year mortality rate among people with advanced prostate cancer remains significant [113]. Finding an effective treatment for advanced BC is an urgent priority because long-term recurrence-free survival is still an unmet need at this stage of the disease [114]. Natural compounds resistant to cancer from many plants can work together. As a result, quercetin (found in onions) and genistein (found in soy) work together to decrease the proliferation of ovarian cancer cells [5].

A study found that tea polyphenols can effectively mitigate FK506-induced cellular apoptosis. The cytotoxicity of FK506 in LLC-PK1 was significantly reduced when treated with a combination of 5 μM (−)-epigallocatechin-gallate (EGCG) and 5 μM of various other compounds. The study also found that the combined administration of EGCG and EGC, EGCG or ECG, and EGC and ECG showed more pronounced synergistic effects in protecting against FK506-induced cell death. Additionally, the combination therapy significantly inhibited heightened intracellular ROS levels 15 min after FK506. The protective properties of green tea extract may be attributed to multiple elements rather than a single ingredient alone [115]. A study reveals that snail and quercetin can enhance the expression of E-cadherin and modulate the Akt/mTOR signaling pathway. Snail inhibition downregulated the ratios of p-Akt/Akt and p-mTOR/mTOR. At the same time, quercetin treatment disrupted the ERK signaling pathway, resulting in a significant reduction in the p-ERK/ERK ratio. These findings suggest that snail and quercetin can affect cellular processes like proliferation, cell cycle regulation, migration, and apoptosis via the Akt/mTOR/ERK signaling pathway. The suppression of snail and/or quercetin effectively suppressed the proliferation, cell cycle advancement, and migration of ccRCC Caki-2 cells, causing apoptosis by regulating E-cadherin, COX2, HIF-1, VEGF/VEGFR2, CD147, and Akt/mTOR/ERK signaling pathways. The study provides new insights into the molecular mechanisms behind these inhibitory effects. It suggests that combining natural products and gene therapy could be an innovative therapeutic approach for renal cancer prevention and treatment [116]. Another study investigates the anti-cancer efficacy of quercetin and hyperoside (QH) in 786-O renal carcinoma cells. The combination showed a significant reduction in ROS production, antioxidant capacity, caspase-3 cleavage, and heightened PARP cleavage. QH also reduced the expression of specificity protein (Sp) transcription factors crucial for cell proliferation, survival, and angiogenesis. The administration of QH led to a drop in Sp1, Sp3, and Sp4 mRNA and protein levels and a significant reduction in survivin production.

Additionally, QH reduced the expression of microRNA-27a (miR-27a) and up-regulated the expression of zinc finger protein ZBTB10, which regulates Sp transcription factors. This suggests that interactions between QH and the miR-27a-ZBTB10 axis may be involved in the downregulation of Sp [117]. Besides, a research group examined the anti-proliferative effect of black tea polyphenol, thearubigin, on human prostate cancer cells. In isolation, thearubigin did not cause any changes in cellular proliferation. Combined with genistein, it suppressed proliferation and triggered cell cycle arrest in the G2/M phase, with dosage affecting the effects. The results suggest combining phytochemicals could be a viable approach to preventing prostate cancer [118].

## Clinical status

Recent changes in clinical studies of genitourinary cancers, such as RCC, prostate adenocarcinoma, and urothelial bladder carcinoma, have led to significant advancements in treatment. Immunotherapy, remarkably immune checkpoint inhibitors, has significantly improved patient survival rates and long-term responses to spreading conditions. Older populations present unique challenges in disease management due to their fragility and illness [119–122]. Diagnosing bladder problems is crucial for Muscle-Invading Urothelial Carcinoma (MIBC) and metastatic disease [123–126]. MIBC is treated with neoadjuvant platinum-based chemotherapy and radical cystectomy, with low mortality and complications [127]. Patients with CPI metastases unsuitable for platinum-based therapy or who have advanced disease after initial treatment can cover platinum. Nivolumab, nivolumab, atezolizumab, pembrolizumab, and durvalumab are some of the CPIs available for those who have completed their treatment with platinum-based chemotherapy [128–132]. The FDA licenses Erdafitinib and genetically engineered FGFR2 or FGFR3 for locally formed or metastatic urothelial carcinomas that can be treated with advanced platinum [133–136]. Renal cell carcinoma is a recurrent genitourinary cancer with over 320,000 annual diagnoses. It resisted radiation, hormone therapy, and conventional chemotherapy [137]. Cytokine-based treatment has moderate results and a poor prognosis. Progressive identification of VHL tumor-suppressing genes and mutations in 90% of patients with uninfected cell RCC has helped explain molecular interactions in tumor microenvironments [138].

The administration of oral isoquercetin could alleviate fatigue among patients with kidney cancer undergoing sunitinib treatment. In light of the insufficient information regarding administering isoquercetin concurrently with sunitinib, a phase I clinical trial was conducted to evaluate the safety of isoquercetin manufactured under Good Manufacturing Practice (GMP) guidelines. The trial involved administering two different dosages of isoquercetin, 450 and 900 mg daily. The study involved 12 patients who were administered isoquercetin alongside 50 mg sunitinib for a median duration of 81 days (with an interquartile range of 75.5 to 86.5 days). There were no instances where the 12 patients necessitated the use of isoquercetin suspension or a reduction in isoquercetin dosage due to adverse events. No anomalies were observed in the lower extremities' electrocardiogram, cardiac, or Doppler ultrasound examinations. The present phase I trial demonstrated that isoquercetin exhibited a notable safety profile and an initial indication of efficacy in ameliorating the adverse events associated with sunitinib [139]. The preventive benefits of curcumin against chemotherapy-induced nephrotoxicity are now the subject of research. It does this by reducing FEN-1 (Flap endonuclease 1) expression, which reduces cisplatin resistance. Curcumin is anticipated to demonstrate its promising results, whether it is paired with tyrosine kinase inhibitors in the treatment of lung cancer, with gemcitabine in the treatment of pancreatic cancer, pre-cancerous lesions in the treatment of cervical cancer, or its function in the treatment of sarcoma [140]. A placebo-controlled, double-blind, randomized clinical study known as NCT03211104 is conducted to investigate whether or not curcumin affects prostate cancer patients' progression with on/off hormone deprivation treatment [141]. Also, many trials are ongoing or completed regarding the anti-cancer role of curcumin [142, 143].

## Concluding remarks and future directions

Targeted genitourinary cancer treatment faces numerous challenges, with few clinical trials completed. Imaging investigations remain crucial for staging, post-treatment follow-up, and diagnosis but have limitations. Progress is being made, and new drugs, particularly immunotherapeutic ones, have significantly impacted this area. Magnetic resonance-guided biopsy may detect significant component analysis in high-risk patients who have failed previous transrectal ultrasound-guided biopsies. However, due to time and high-cost constraints, extensive research is needed to evaluate its efficacy in clinical practice. Although new therapeutic approaches may not be practical for every patient due to their uniqueness, biomarkers, and related genes are crucial to consider when developing specialized drugs. The drug is likely to become more widely available in clinical settings, drugs are likely to become more widely available. Researchers have identified potential cancer therapeutic targets, such as flavonoids, which can inhibit cancer cell proliferation, migration, and invasion while promoting apoptosis. Small molecules, including flavonoids, can be used to treat cancer, but further studies are needed to demonstrate their efficacy in treating genitourinary cancer. Future studies revealing the combined effects of curcumin, resveratrol, paclitaxel, or their derivatives on cancer may lead to new therapeutic applications.

## Data Availability

The datasets generated during and/or analyzed during the current study are available from the corresponding author upon reasonable request.

## Abbreviations

MBBC: Metastatic bladder cancer
FDA: Food and Drug Administration
RCC: Renal cell carcinomas
VHL: Von Hipple-Lindau
PSCC: Penile squamous cell carcinoma
PUC: Plasmacytoid urethral carcinoma
BC: Bladder cancer
HPV: Human papillomavirus
VEGF: Vascular endothelial growth factor
BCC: Bladder cancer cell
NF-κB: Nuclear factor-kappa B
IAP: Inhibitors of apoptosis protein
ROS: Reactive oxygen species
EMT: Epithelial–mesenchymal transition
TRX1: Transient receptor potential channel 1
OXPHOS: Oxidative phosphorylation process
MIBC: Muscle-invading urothelial carcinoma
MIF: Macrophage migration inhibitory factor
RCA: Renal-cell adenoma
